# Study on the diagnostic value of MDCT extramural vascular invasion in preoperative N staging of gastric cancer patients

**DOI:** 10.1186/s12880-024-01200-z

**Published:** 2024-01-19

**Authors:** Zhengqi Zhu, Mimi Mao, Anyi Song, Haipeng Gong, Jianan Gu, Yongfeng Dai, Feng Feng

**Affiliations:** Department of Radiology, Nantong Tumor Hospital, No. 30, Tongyang North Road, Nantong, Jiangsu Province 226006 China

**Keywords:** Extramural vascular invasion, Gastric cancer, N staging, Diagnostic value

## Abstract

**Background:**

To explore the diagnostic value of multidetector computed tomography (MDCT) extramural vascular invasion (EMVI) in preoperative N Staging of gastric cancer patients.

**Methods:**

According to the MR-defined EMVI scoring standard of rectal cancer, we developed a 5-point scale scoring system to evaluate the status of CT-detected extramural vascular invasion(ctEMVI), 0–2 points were ctEMVI-negative status, and 3–4 points were positive status for ctEMVI. Patients were divided into ctEMVI positive group and ctEMVI negative group. The correlation between ctEMVI and clinical features was analyzed. Receiver operating characteristic (ROC) curve was used to evaluate the diagnostic efficacy of ctEMVI for pathological metastatic lymph nodes and N staging, The sensitivity, specificity, accuracy, positive predictive value (PPV), and negative predictive value (NPV) of pathological N staging using ctEMVI and short-axis diameter were generated and compared.

**Results:**

The occurrence rate of lymphovascular invasion (LVI) and proportion of tumors with a greatest diameter > 6 cm in the ctEMVI positive group was higher than that in the ctEMVI negative group (*P* < 0.05). Spearman correlation analysis showed a positive correlation between ctEMVI and LVI, N stage, and tumor size (*P* < 0.05). For ctEMVI scores ≥ 3,The AUC of ctEMVI for diagnosing lymph node metastasis, N stage ≥ N2, and N3 stage were 0.857, 0.802, and 0.758, respectively. The sensitivity, NPV and accuracy of ctEMVI for diagnosing N stage ≥ N2 were superior to those of short-axis diameter (*P* < 0.05), while sensitivity, specificity, PPV, NPV, and accuracy of ctEMVI for diagnosing N3 stage were superior to those of short-axis diameter (*P* < 0.05).

**Conclusion:**

ctEMVI has important value in diagnosing metastatic lymph nodes and advanced N staging. As an important imaging marker, ctEMVI can be included in the preoperative imaging evaluation of patients, providing important assistance for clinical guidance and treatment.

## Background

Gastric cancer is a common malignant tumor in the digestive system, and its early clinical symptoms are often hidden, so patients are often diagnosed with advanced gastric cancer, and the clinical outcome is usually poor [1]. Lymph node metastasis is one of the most common clinical manifestations of gastric cancer, and is often discovered when patients seek medical attention [2]. The N staging of gastric cancer in the AJCC eighth edition TNM staging system is based on the number of metastatic lymph nodes [3]. Study has shown that N staging is an important factor affecting the clinical prognosis of gastric cancer because there is a significant difference in the 5-year overall survival rate among different N stages [4]. Preoperative determination of the N stage of gastric cancer and neoadjuvant chemotherapy (NAC) for patients with advanced N staging can improve clinical prognosis [5].

Extramural vascular invasion (EMVI) is a common complication of gastric cancer, which refers to the invasion of tumor cells into the venous vessels outside the muscular layer [6]. MR-detected extramural vascular invasion (mrEMVI) has been widely studied in rectal cancer. Study has shown that mrEMVI is an independent predictor of poor clinical outcomes in patients and is associated with high tumor recurrence and metastasis rates [7]. However, MRI is expensive, takes a long scanning time, is easily affected by stomach peristalsis, and is currently difficult to use as a routine preoperative imaging examination for gastric cancer patients. Multidetector computed tomography(MDCT), as a routine imaging examination for preoperative gastric cancer patients, has high-resolution images and multi-planar reconstruction techniques, which can accurately identify EMVI before surgery [8]. Yang et al. [9] found that CT-detected EMVI was significantly associated with positive lymph node status. Gao et al. [10] investigated the molecular mechanism underlying CT-detected EMVI and constructed a CT-detected EMVI-related gene model which could be used to predict the overall survival in gastric cancer patients. Currently, only a few studies have reported the importance of CT-detected extramural vascular invasion(ctEMVI) in preoperative radiological evaluation of gastric cancer. Therefore, this study aims to investigate the diagnostic value of ctEMVI in preoperative N staging of gastric cancer patients.

## Methods

### Research object

The study was approved by the Ethics Committee of Nantong Tumor Hospital (No.2023-A 07) and were performed in accordance with World Medical Association (WMA) Declaration of Helsinki. We Retrospectively analyzed 200 cases of gastric cancer patients confirmed by surgical pathology in our hospital from January 2020 to June 2022. Inclusion criteria: [1] radical gastrectomy and D2 lymph node dissection within 2 weeks after CT scan; [2] no previous gastrectomy; [3] no history of NAC; [4] visible lesions on CT images; [5] all patients have complete preoperative clinical imaging data and postoperative pathological data. Seventy-five patients were excluded from the study for the following reasons: having received other treatments before gastrectomy (*n* = 22), nonvisible gastric cancer on CT (*n* = 20), the CT scan was performed more than 2 weeks before gastrectomy (*n* = 25), and no availability of preoperative water-distended stomach (*n* = 8). In the end, 125 patients were included in this study.

### Instruments and methods

Siemens SOMATOM Sensation 64 CT scanner, with a tube voltage of 120 kV and automatic tube current modulation. The slice thickness and slice interval are both 5 mm, with a pitch of 1.0, matrix of 512 × 512, and collimation of 1.0 mm. Contrast agent iohexol (GE Healthcare, Shanghai, China) (300 mgI/ml, 1.5 ml/kg) was injected intravenously at a flow rate of 2.5-3.0 ml/s for enhanced scanning, with arterial and venous phase images acquired at 25 and 50 s delays, respectively. All images were transferred to the Syngo.via VB20 workstation (Siemens Healthineers, Forchheim, Germany) for reconstruction in coronal, sagittal, and transverse planes (with a reconstructed slice thickness of 1.0 mm).

### Image analysis

By two senior radiologists, without knowing the pathology results, the images were observed and analyzed, including venous phase transverse, sagittal and coronal reconstructed images with a reconstructed slice thickness of 1.0 mm. A third senior radiologist was asked to review the images and make the final diagnosis if there was a controversy between the two radiologists.

**Fig. 1:**
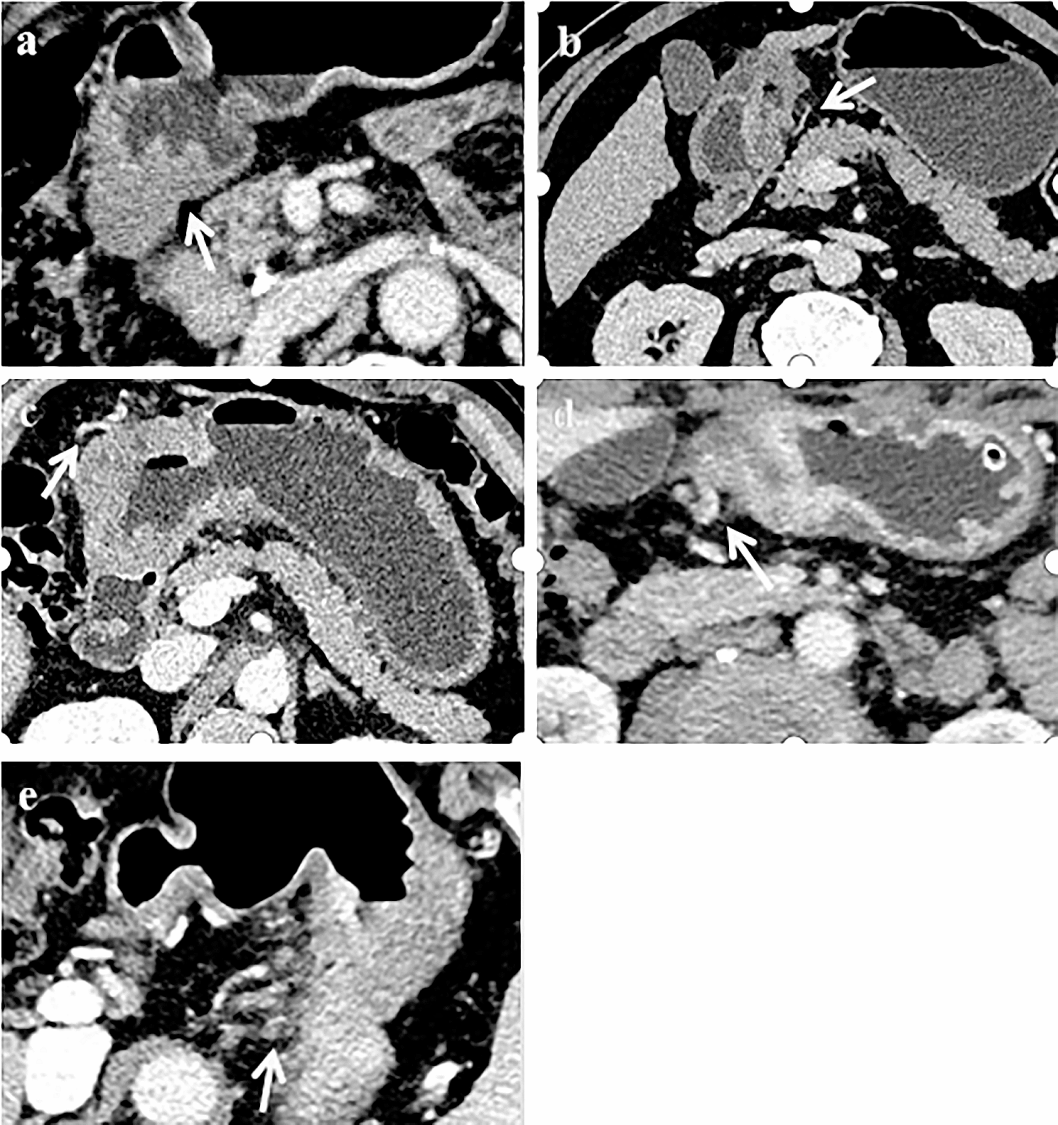
MDCT transverse section image of venous phase. (a) 0 point: The mass penetrated the gastric wall, and there was no blood vessel adjacent to the tumor lesion (white arrow). (b) 1 point: The mass extended slightly outwards through the gastric wall, but the adjacent blood vessel is not involved (white arrow). (c) 2 point: The mass penetrated the gastric wall, with blood vessels around the tumor lesion, but vascular lumen was normal and there was no tumor density shadow inside the lumen (white arrow). (d) 3 point: The mass presented as nodular extension to the blood vessels surrounding the stomach, with slight dilation of the affected vessels and filling defects seen within (white arrow). (e) 4 point: The mass presented as multiple nodules extending into the blood vessels surrounding the stomach, with significant dilation and irregular morphology of the affected vessels, and filling defects seen within (white arrow)

According to the EMVI scoring criteria defined by high-resolution MR images of rectal cancer [11], a 5-point scale scoring system was used to evaluate the status of ctEMVI, 0–2 points were negative for ctEMVI, and 3–4 points were positive for ctEMVI (Fig. 1). The diagnostic criterion for metastatic lymph nodes on MDCT imaging was a lymph node short-axis diameter exceeding 8 mm [12]. According to the eighth edition of the AJCC TNM staging system, MDCT nodal staging was categorized into stages N0 through N3. The greatest diameter of the tumor was measured on the transverse CT image as the size of the tumor.

### Pathological analysis

The histopathological characteristics of gastric cancer were evaluated by two experienced gastrointestinal pathologists, and disagreements were resolved through consultation. The degree of tumor differentiation and the presence of lymphovascular invasion (LVI) were recorded. Based on the results of pathological examinations, lymph nodes were determined as metastatic or not, and the number of metastatic lymph nodes was recorded. The pathological N staging was evaluated according to the AJCC 8th edition gastric cancer TNM staging system.

### Statistical analysis

Quantitative data that conforms to the normal distribution was represented by the mean ± SD, and independent sample t-tests were used for comparison between two groups; count data was represented by percentages (%), and the χ^2^ test was used for comparison between two groups. Spearman correlation analysis was used to evaluate the correlation between ctEMVI and clinicopathological indicators. Receiver operating characteristic (ROC) curve was used to evaluate the diagnostic efficacy of ctEMVI for diagnosing pathological metastatic lymph nodes and N staging. Kappa consistency test was used to evaluate the consistency between observers in ctEMVI and short-axis diameter. Using pathological N staging as the gold standard, the accuracy, sensitivity, specificity, positive predictive value (PPV), and negative predictive value (NPV) of short-axis diameter and ctEMVI diagnosis of N staging were calculated respectively. Consider a statistical significance if *P* is less than 0.05.

## Results

### General information

This study included a total of 125 patients, including 87 males and 38 females, aged between 37 and 88 years with a mean age of (63.2 ± 10.6) years. Based on the ctEMVI score, the positive group included 77 cases and the negative group included 48 cases. The evaluation of ctEMVI and short-axis diameter showed excellent inter-observer agreement (with Kappa values of 0.841 and 0.852, respectively).

### Comparison of clinicopathological features between two groups

The rate of LVI was higher in the ctEMVI positive group compared to the ctEMVI negative group [79%(61/77) vs. 33%(16/48), (*P* < 0.001)]. The proportion of tumors with a greatest diameter > 6 cm was higher in the ctEMVI positive group [73%(56/77) vs. 30%(14/48), (*P* < 0.001)]. Age, tumor location, and differentiation degree did not show any statistical significance (*P* > 0.05) (Table 1).

**Table 1:** Comparison of clinical characteristics between ctEMVI positive group and ctEMVI negative group

	ctEMVI positive (*n* = 77)	ctEMVI negative(*n* = 48)	t/χ^2^ value	P value
Age(year)	64.6 ± 10.14	61.1 ± 10.71	-1.846	0.067
Sex			0.027	0.870
Male	54(70%)	33(69%)		
Female	23(30%)	15(31%)		
Tumor location			3.287	0.193
Fundus	15(19%)	4(8%)		
Gastric body	25(33%)	15(31%)		
Gastric antrum	37(48%)	29(61%)		
Differentiation degree			5.098	0.078
Low	48(62%)	21(44%)		
Medium	27(35%)	20(42%)		
High	2(3%)	7(14%)		
N staging			42.010	< 0.001
N0	5(6%)	26(54%)		
N1	14(18%)	10(21%)		
N2	20(26%)	9(19%)		
N3	38(50%)	3(6%)		
LVI	61(79%)	16(33%)	26.321	< 0.001
Tumor size (cm)			22.770	< 0.001
< 6	21(27%)	34(70%)		
> 6	56(73%)	14(30%)		

### Analysis of the correlation between ctEMVI and clinicopathological features

**Fig. 2:**
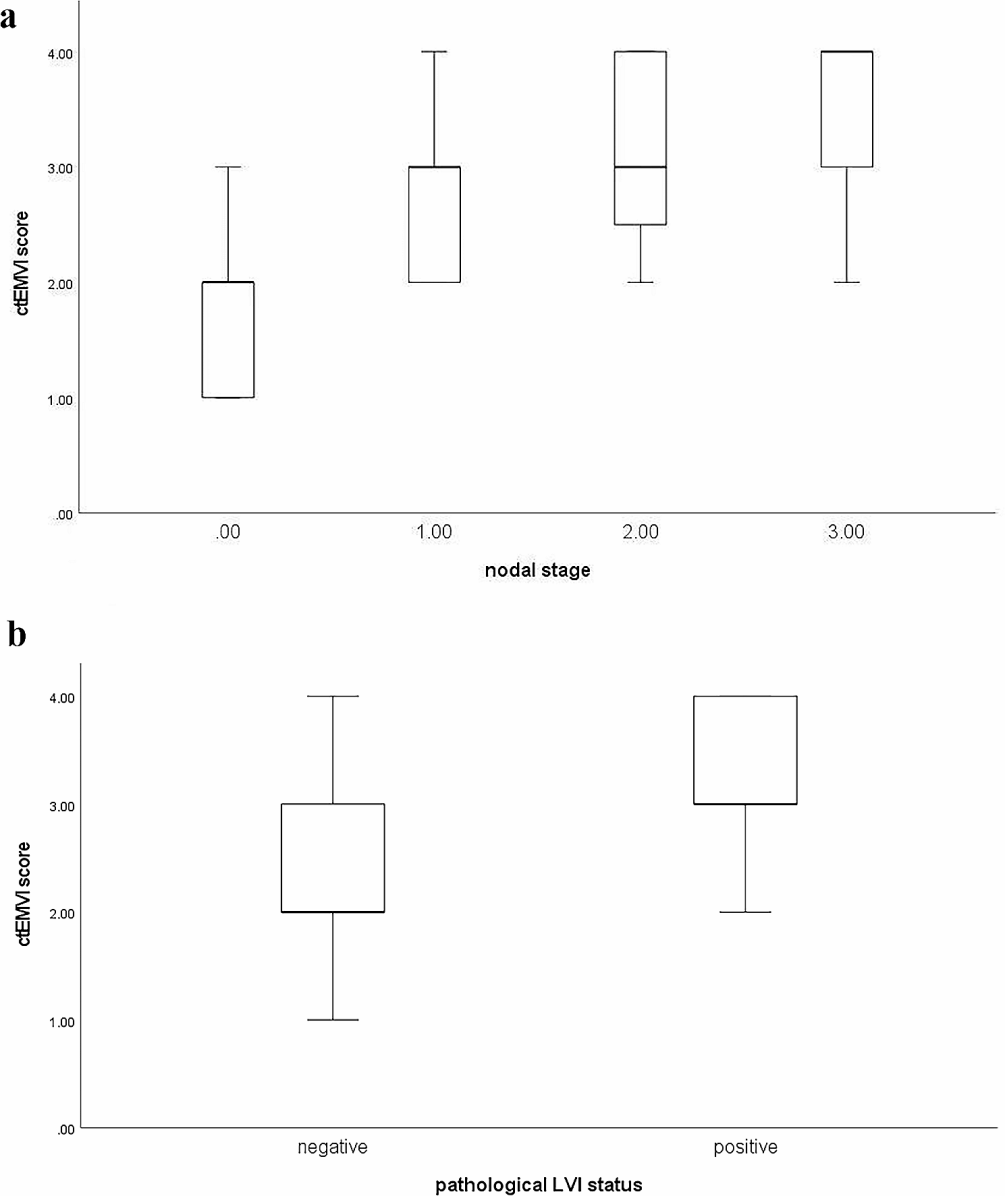
(a) Boxplots showed the significant differences of ctEMVI score between pathological nodal stage (N0vsN1vsN2vsN3) (P < 0.001). (b) Boxplots showed the significant differences of ctEMVI score between different pathological LVI status. (P < 0.001)

The ctEMVI score between different N staging and LVI status showed statistical differences (*P* < 0.001) (Fig. 2). Spearman correlation analysis showed a positive correlation between ctEMVI and LVI, N staging, and tumor size(*r =* 0.478, *P* < 0.001; *r =* 0.581, *P* < 0.001; *r* = 0.424, *P* < 0.001).

### The diagnostic efficacy of ctEMVI on pathological metastatic lymph nodes and N staging

**Fig. 3:**
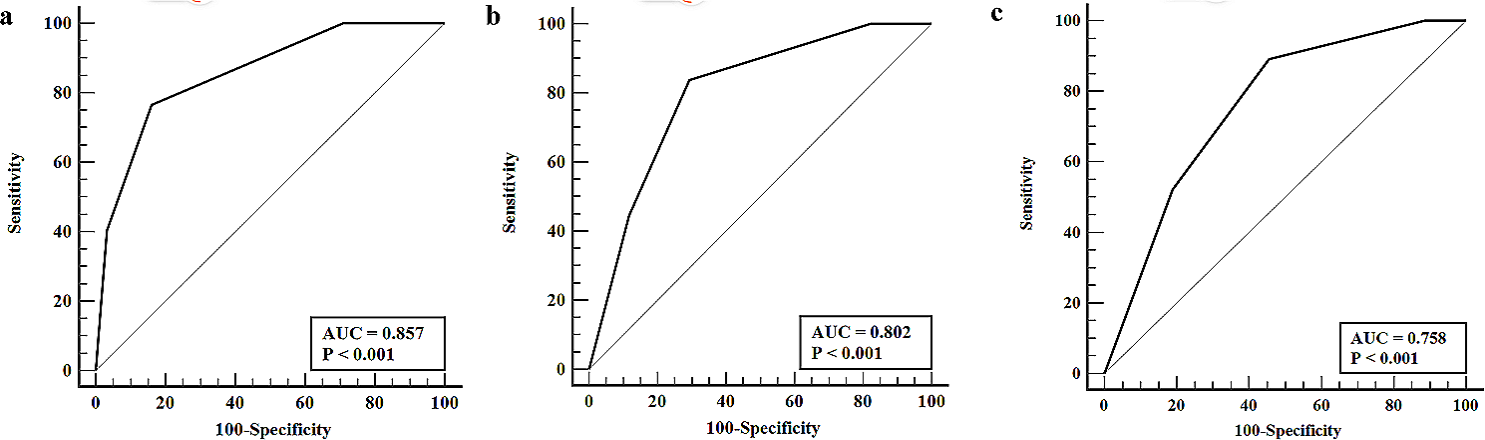
Receiver operating characteristic (ROC) curve. (a) ROC curve of ctEMVI in the diagnosis of metastatic lymph nodes. (b) ROC curve of ctEMVI in the diagnosis of N stage ≥ N2. (c) ROC curve of ctEMVI in diagnosis of N3 stage

ROC curve analysis results showed that the AUC value of ctEMVI in diagnosing metastatic lymph nodes was 0.857, when the ctEMVI score of 3 or greater, the sensitivity was 76% and the specificity was 84% (Fig. 3A). The AUC value of ctEMVI in diagnosing N stage ≥ N2 was 0.802, when the ctEMVI score of 3 or greater, the sensitivity was 83% and the specificity was 65% (Fig. 3B). The AUC value of ctEMVI in diagnosing N3 stage was 0.758, when the ctEMVI score of 3 or greater, the sensitivity was 92% and the specificity was 53% (Fig. 3C).

### Comparison of diagnostic efficacy of ctEMVI and short-axis diameter for pathological N staging

The sensitivity, NPV, and accuracy of ctEMVI diagnosis for N stage ≥ N2 are superior to those of short-axis diameter, and the differences have statistical significance (*P* < 0.05); the sensitivity, specificity, PPV, NPV and accuracy of ctEMVI diagnosis for N3 stage are also superior to those of short-axis diameter, and the differences have statistical significance (*P* < 0.05) (Table 2).

**Table 2:** Comparison of diagnostic efficacy between ctEMVI and short-axis diameter for N stage ≥ N2 and N3 stage

		Sensitivity	Specificity	PPV	NPV	Accuracy
N stage ≥N2	Short-axis diameter	33%(23/70)	74%(41/55)	62%(23/37)	46%(46/88)	52%(64/125)
	ctEMVI	83%(58/70)	65%(36/55)	75%(58/77)	75%(36/48)	75%(94/125)
*P*		< 0.001	0.298	0.147	0.01	< 0.001
N3 stage	Short-axis diameter	21%(9/43)	71%(58/82)	27%(9/33)	63%(58/92)	54%(67/125)
	ctEMVI	93%(38/41)	54%(45/84)	49%(38/77)	94%(45/48)	66%(83/125)
*P*		< 0.001	0.036	0.024	< 0.001	0.039

## Discussion

This study aims to explore the diagnostic value of ctEMVI in the preoperative N staging of gastric cancer patients. The results show that there is a correlation between ctEMVI and preoperative N staging of patients. Compared with short-axis diameter, ctEMVI exhibits higher diagnostic value in diagnosing N stage ≥ N2 and N3 stage. making it an important imaging biomarker for the preoperative N staging of gastric cancer patients.

In our study, ctEMVI was positively correlated with pathological LVI status. Compared with the negative LVI status, the positive LVI status has higher ctEMVI score. EMVI refers to the invasion of tumor cells into veins beyond the muscularis propria, while LVI refers to the invasion of lymphatic vessels and capillaries in the submucosa or muscularis layer by the tumor. These two are fundamentally different [13]. However, lymphatic and vascular pathways serve as interconnected routes for cancer cell dissemination [14], so EMVI often co-occurs with LVI. It was also found in our study that there was a positive correlation between ctEMVI and tumor size. The tumor size of ctEMVI positive group was significantly larger than the ctEMVI negative group. This may be due to the fact that EMVI mainly spreads along neurovascular bundles in tumor tissue, and the larger the tumor volume, the greater the contact area with blood vessels, leading to a higher risk of EMVI [15].

Previous study has shown that ctEMVI was an independent factor that affects lymph node metastasis in gastric cancer [9]. The ROC curve analysis in our study showed that when the ctEMVI score was 3 or greater, the AUC for diagnosing lymph node metastasis was 0.857, with a sensitivity of 76% and a specificity of 83%. Our result also suggests that ctEMVI has important value in diagnosing lymph node metastasis in gastric cancer. This indicates that EMVI, as a way for tumors to spread along nerve blood vessels, does not occur in isolation and is often accompanied by lymphatic invasion around nerve blood vessels [16]. Our study also explored the relationship between ctEMVI and the severity of lymph node metastasis, known as N staging. The results showed that ctEMVI score between different N staging has statistical difference. The ctEMVI was positively correlated with N staging. Wang et al. [8] also found a positive correlation between ctEMVI and N staging with the highest incidence of positive ctEMVI in N3 stage gastric cancer, which is consistent with our study.

Our study used a short-axis diameter of lymph nodes greater than 8 mm as the MDCT nodal staging standard. In terms of diagnosing N stage ≥ N2 and N3 stage, the sensitivity was 32% and 20%, the specificity was 74% and 70%, and the accuracy was 52% and 53%, which is consistent with previous studies [17]. Yan et al.‘s study [18] showed that MDCT has low sensitivity in predicting N2/N3 stage cancer, and the difference in lymph node counts between MDCT and pathological evaluation increases with increasing N staging, resulting in inevitably low N staging by MDCT evaluation. The limitation of only using lymph node diameter to determine metastatic lymph nodes is that smaller lymph nodes can still metastasize, so new imaging markers are needed to overcome the limitations of low sensitivity in diagnosing advanced N staging by traditional MDCT nodal staging. The ROC curve of our study showed that when the ctEMVI score was 3 or greater, the AUC for diagnosing N stage ≥ N2 was 0.802, the sensitivity was 83%, and the specificity was 65%. The AUC for diagnosing N3 stage was 0.758, the sensitivity was 93%, and the specificity was 54%. Compared with short-axis diameter, ctEMVI has higher sensitivity, NPV and accuracy in diagnosing N stage ≥ N2, and higher sensitivity, specificity, PPV, NPV and accuracy in diagnosing N3 stage. ctEMVI can be an important imaging marker for diagnosing advanced N staging and overcoming the limitation of low sensitivity of traditional MDCT nodal staging. Our study also found that ctEMVI has moderate specificity in diagnosing advanced N staging, which indicates that lymph node metastasis is a complex process where tumor cells can spread to lymph nodes through blood vessels or other pathways, such as lymphatic channels [19].

The N staging is one of the important factors affecting the overall survival rate of patients. Bando et al. [20] analyzed the survival curves of the three (cN0-N1-N2/3) and four (cN0-N1-N2-N3) cN groups and found that the N2/3 and N3 stage have a lower 5-year overall survival rate. Surgical treatment be preceded by neoadjuvant chemotherapy is recommended for advanced stage gastric cancer in the Western countries [21]. A study of nodal status in the Perioperative Chemotherapy versus Surgery Alone for Resectable Gastroesophageal Cancer (MAGIC) trial demonstrated that people who received neoadjuvant chemotherapy with a regimen of epirubicin, cisplatin, and fluorouracil tend to have less advanced nodal disease (N0 or N1) and a significantly higher likelihood of overall survival [22]. Wu et al. [23] found that nodal downstaging is an important hallmark representing the effectiveness of NAC for GC and positively impact the survival of these patients. Therefore, the inclusion of ctEMVI in the preoperative imaging assessment will help to identify patients with high risk of advanced N staging who truly need and could most benefit from NAC.

This study still has limitations: (1) The selected patients included early and advanced gastric cancer patients, which may lead to potential confounding factors; (2) The specificity of ctEMVI in determining advanced N staging is general, and future exploration of multiple imaging indicators combined use is needed to improve the diagnostic efficacy of N staging; (3) There is no pathological evidence supporting ctEMVI in this study, and further research is still needed.

In conclusion, there is a correlation between ctEMVI and preoperative N staging in patients, ctEMVI has important value in diagnosing metastatic lymph nodes and advanced N staging. As an important imaging marker, ctEMVI can be included in preoperative imaging evaluation of patients, providing important assistance for clinical guidance and treatment.

## Data Availability

The corresponding author can provide the datasets used or analyzed in the current study upon reasonable request.
